# Tunable Wettability of Biodegradable Multilayer Sandwich-Structured Electrospun Nanofibrous Membranes

**DOI:** 10.3390/polym12092092

**Published:** 2020-09-15

**Authors:** A. K. M. Mashud Alam, Elena Ewaldz, Chunhui Xiang, Wangda Qu, Xianglan Bai

**Affiliations:** 1 Department of Apparel, Events, and Hospitality Management, Iowa State University, Ames, IA 50011, USA; mashud@iastate.edu; 2 School of Materials Science and Engineering, Georgia Institute of Technology, Atlanta, GA 30332, USA; eewaldz@gatech.edu; 3 Department of Mechanical Engineering, Iowa State University, Ames, IA 50011, USA; wqu@iastate.edu (W.Q.); bxl9801@iastate.edu (X.B.)

**Keywords:** tunable wettability, surface energy, wetting envelope, regenerated cellulose, poly (lactic acid)

## Abstract

This research aims to develop multilayer sandwich-structured electrospun nanofiber (ENF) membranes using biodegradable polymers. Hydrophilic regenerated cellulose (RC) and hydrophobic poly (lactic acid) (PLA)-based novel multilayer sandwich-structures were created by electrospinning on various copper collectors, including copper foil and 30-mesh copper gauzes, to modify the surface roughness for tunable wettability. Different collectors yielded various sizes and morphologies of the fabricated ENFs with different levels of surface roughness. Bead-free thicker fibers were collected on foil collectors. The surface roughness of the fine fibers collected on mesh collectors contributed to an increase in hydrophobicity. An RC-based triple-layered structure showed a contact angle of 48.2°, which is comparable to the contact angle of the single-layer cellulosic fabrics (47.0°). The polar shift of RC membranes on the wetting envelope is indicative of the possibility of tuning the wetting behavior by creating multilayer structures. Wettability can be tuned by creating multilayer sandwich structures consisting of RC and PLA. This study provides an important insight into the manipulation of the wetting behavior of polymeric ENFs in multilayer structures for applications including chemical protective clothing.

## 1. Introduction

Surface wettability is one of the most critical considerations in designing membranes for many functional applications, such as liquid separation, fluid control, corrosion protection, anti-fouling surfaces, self-cleaning materials, and protective textiles [1]. Traditionally, the contact angle from Young’s equation is the most widely used technique to evaluate the wettability of a solid surface [2,3]. The static contact angle is determined from the surface energies of the associated solid and liquid, based on the assumption that a chemically homogeneous surface is totally smooth [4]. The wettability is generally low when the total surface energy of the solid surface is low, and hence the contact angle is high. Wetting models by Wenzel and Cassie-Baxter provided further clarification of the mechanism of wettability and introduced surface roughness and surface heterogeneity as the two critical factors for surface wetting. The chemical heterogeneity and surface roughness of a real surface presents a multiplicity of local contact angles, metastable states, and hysteresis effects [5]. Therefore, to be able to capture the metastable states, dynamic contact angle measurements are an advantageous tool for the evaluation of the contact angle, surface free energy (SFE), or wettability. Among all the thermodynamic approaches (i.e., Zisman, Fowkes, Owens–Wendt, Wu, Equation of State, Acid-base, etc.) to the calculation of SFE of a solid surface, the Owens–Wendt–Rable–Kaeble (OWRK) model has been one of the most commonly used methods for moderately polar low surface energy polymeric surfaces [6]. This method considers the total SFE of a solid surface as a sum of all interactions at the solid/liquid interface and divided into two components, namely the dispersion component and polar component.

Among the strategies employed throughout industries for modifying wettability, the manipulation of the surface energy and surface roughness of thermoplastic polymers has been immensely studied. Numerous techniques, including layer-by-layer assembly, the sol-gel process, dip coating, spray coating, plasma treatment, and electrospinning, have been employed for manipulating the wettability of the polymeric surfaces [7,8]. Although the commonly employed strategy of manipulating surface roughness through a coating of nanoparticles of fluoropolymers, SiO_2_, TiO_2_, and ZnO, on the membrane surface was found to be successful, concerns exist about its stability and durability, as well as the potential toxicity to the environment [1,9]. Therefore, this study is designed, for the first time, solely based on the ENF membranes of biodegradable polymers in multilayer structures without any toxic surface treatment. The resulting novel lightweight electrospun structure can be employed for chemical protective clothing (CPC) for pesticide applicators, who risk their lives due to the toxic effects of pesticides to avoid bulky CPC.

Electrospinning is one of the most popular techniques employed to fabricate ENF membranes with tunable diameters with a highly interconnected porous network where fiber properties such as morphology, topography, architecture, and porosity can be engineered by manipulating the solution parameters and process parameters, including the feeding rate, applied voltage, collection distance, and collector type [10,11]. Electrospun fibers, among the structures studied so far (i.e., fibers, films, and molds), are considered superior for membrane applications to shift the hydrophobicity of materials due to their nanoscale diameters, large surface area to volume ratio, increased surface roughness, and porous structures [12,13,14]. The variations in ENF properties, including surface roughness and porosity, introduce a deviation in surface energy, which results in a variation of the wettability of the surfaces according to the Cassie and Baxter model [7]. These electrospun membranes for wettability manipulation are popularly constructed of petroleum-based synthetic hydrophobic materials, including polypropylene (PP), polyvinylidene fluoride (PVDF), polyurethane (PU), and polytetrafluoroethylene (PTFE). Therefore, the development of electrospun membranes based on biodegradable materials is of great significance [15].

As an attractive alternative to petroleum-based polymers, biodegradable polymers such as polyvinyl alcohol (PVA), polyvinyl acetate (PVAc), polycaprolactone (PCL), polylactic acid (PLA), polyethylene glycol (PEG), cellulose derivatives, sodium alginate, chitin, chitosan, collagen, etc. have currently been receiving considerable scientific and technical interest due to their biocompatibility and ecological advantages [15,16,17]. Among them, cellulose acetate (CA) and poly (lactic acid) (PLA) are highly attractive owing to their incredible polymer properties. CA is one of the most important biodegradable polymers produced from natural cellulosic materials such as wood and cotton linters. CA is a raw material that can be transformed into regenerated cellulose (RC) via hydrolysis treatment. Due to the presence of a high amount of hydroxyl group, RC is highly biocompatible and excessively wettable. However, the excessive wettability of RC sometimes limits its widespread application [18]. On the other hand, PLA, a hydrophobic thermoplastic aliphatic polyester with an excellent resiliency, elastic recovery, and sunlight resistance, and derived from naturally renewable resources such as corn starch, is considered as one of the more promising and sustainable fiber materials [19,20]. Recently PLA has been studied for various applications, including for the controlled release of drugs [6], as an effective oil sorbent [21], and as a biosensor [22]. However, the low tensile strength, brittleness, and low wettability have been limiting factors that hindered the widespread application of PLA [22]. Recently, Chen and fellow researchers employed RC to reinforce the PLA in a PLA/RC scaffold via electrospinning in combination with freeze-drying [17]. However, a PLA/RC-based all-electrospun structure for wettability manipulation is rarely found in the literature and needs to be explored [17].

In this work, two different biodegradable polymers (CA and PLA) were employed to develop multilayer sandwich-structured ENF membranes. Two different copper collectors, including copper foil and 30-mesh copper gauze, were employed to examine the effect of collector geometry on the surface properties of ENF membranes. Scanning electron microscopy (SEM), Fourier transform infrared spectrometer (FT-IR), Instron^®^ tensile tester, and video-based dynamic contact angle analyzer were used to characterize the ENF structures. Multilayered sandwiched structures were fabricated with RC and PLA ENFs to manipulate the wettability to attain specific wetting behaviors. The effects of the collector types and layered structures on the contact angle, surface energy, and wettability were examined. The results can facilitate the prediction of the manipulation of the wetting behavior of the solid surfaces by liquids to which the surface might be exposed to during real field applications such as chemical protective clothing for pesticide applicators.

## 2. Materials and Methods 

### 2.1. Materials

Poly (lactic) acid, Ingeo™ biopolymer 6201D, was purchased from Nature Works (Minnetonka, MN, USA) in pellet form. The PLA thermoplastic fiber-grade resin was derived from annually renewable resources with specific gravity of 1.24, glass transition temperature (T_g_) of 55–60 °C, and crystalline melting temperature (T_m_) of 150–170 °C (according to the supplier datasheet). The weight-average molecular weight and polydispersity index, adopted from the corresponding author’s previous research findings, determined by Size Exclusion Chromatography (SEC) was 143 kDa and 1.8, respectively [23]. Cellulose acetate (CA) with a 39% acetyl content and number average molecular weight ($\bar{M_{n}}$) of 30 kDa, determined by Gel Permeation Chromatography (GPC), was purchased from Sigma-Aldrich (St. Louis, MO, USA). Sodium hydroxide Redi-Dri™ anhydrous ACS reagent grade (≥97%) pellets were also purchased from Sigma-Aldrich. Certified ACS grade chloroform, acetone, and glycerol with a ≥99.9% assay were purchased from Fisher Chemical (Fair Lawn, NJ, USA). Ultrapure deionized formamide was purchased from VWR life sciences (Radnor, PA, USA). Additionally, 300 mm × 300 mm copper gauzes (30-mesh), made with 0.15 mm diameter copper wires, were purchased from Alfa Aesar (Ward Hill, MA, USA). Copper foil (annealed, 99.8% metal basis) with a 0.05 mm thickness was also purchased from Alfa Aesar.

### 2.2. Methods

#### 2.2.1. Preparation of Electrospinning Solution 

CA solution at 15% (*w*/*v*) was prepared by dissolving CA overnight in acetone under constant stirring with a Burrell wrist-action^®^ shaker, model-75 (Burrell Scientific LLC, Pittsburgh, PA, USA), to obtain a homogeneous solution. For PLA electrospinning, an 8 wt % solution was prepared by dissolving PLA pellets in a binary solvent system comprised of chloroform and acetone in a 1:3 ratio (*v*/*v*) of acetone to chloroform.

#### 2.2.2. Electrospinning 

A freshly prepared solution was loaded into a 10 mL plastic syringe attached to a stainless-steel needle with an inner diameter of 0.8 mm. The solution was then continuously fed at a fixed rate of 0.05 mL min^−1^ by a syringe pump (Harvard Apparatus, Holliston, MA, USA). A fixed high voltage of 25 kV was applied between the needle tip and the collector by a DC power supply instrument (Gamma High Voltage Research, Ormond Beach, FL, USA). The rotating drum collector was placed at a distance of 12 cm from the needle tip to the collector. The fibers were collected on collectors with different types of surfaces, including copper foil and 30-mesh copper gauzes. The electrospinning process was conducted for 2 h under ambient conditions. Table 1 shows the experimental design used for electrospinning.

#### 2.2.3. Deacetylation of CA Membranes

Deacetylation was performed to remove the acetyl groups from CA membranes following previously reported methods [24]. A deacetylation solution comprising of 4:1 NaOH:EtOH was made using 0.1 M of NaOH in Deionized (DI) water. CA membranes sandwiched between glass fiber mesh were soaked in the deacetylation solution in a glass bowl. A magnetic stirring bar placed on the mesh was kept rotating at 150 rpm for 30 h at room temperature on a hot plate. The membranes were then thoroughly rinsed with deionized water to complete neutralization, which was confirmed by pH paper. They were then air-dried overnight, followed by vacuum drying at 60 °C for 24 h to obtain RC membranes. 

#### 2.2.4. Creation of Multilayer Sandwich-Structures

**Figure 1:**
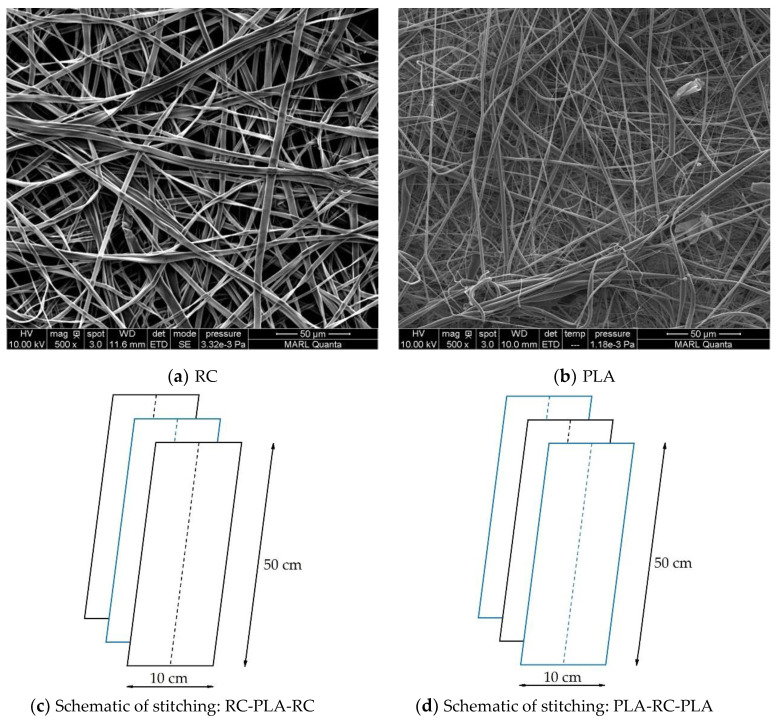
Construction of 3-layered ENF sandwich structures. (**a**) RC-based structure (RC-PLA-RC), (**b**) PLA-based structure (PLA-RC-PLA), and (**c**) schematic of stitching.

Monolayer ENF membranes collected on the same type of collectors were cut in rectangular pieces of 50 mm × 10 mm size and hand-stitched with PLA filament fiber (Figure 1). For example, both PLA and RC membranes collected on copper foil were stitched together to create a 2-layer structure copper foil collector. Similarly, a PLA membrane collected on copper foil was sandwiched between 2 layers of cellulose membranes (collected on copper foil) to create a 3-layer RC-based (RC-PLA-RC) composite structure (Figure 1). For the sake of simplicity of the design, membranes from different collectors were not stitched together. The details of the creation of multilayer structures are summarized in Table 2.

#### 2.2.5. Confirmation of the Development of RC 

The development of RC from CA was studied through a functional group analysis with a Thermo Scientific Nicolet iS10 Fourier transform infrared (FT-IR) spectrometer (Thermo Fisher Scientific Inc., Waltham, MA, USA) equipped with a smart iTR accessory. All the ENFs were dried overnight in an Isotemp^®^ (Model 282A) programmable vacuum oven (Fisher Scientific, Waltham, MA, USA) at room temperature before FT-IR analysis. Each sample was scanned 32 times at a resolution of 4 cm^−1^ and an interval of 1 cm^−1^ over wavenumbers ranging from 750 to 4000 cm^−1^.

#### 2.2.6. Morphology

A field emission scanning electron microscope (FEI Quanta 250, ThermoFisher Scientific, Waltham, MA, USA) was employed to study the size and surface morphology of the ENF membranes. The membranes were kept under vacuum overnight to evaporate any residual solvent or moisture. They were then sputter-coated with a 5 nm layer of iridium to improve the conductivity of the samples for improved imaging. Image J software (National Institute of Health, Bethesda, MD, USA) was used to calculate the diameter and distribution of the fibers. The average and distribution of diameters were determined by measuring 50 representative fibers from the SEM images.

#### 2.2.7. Tensile Properties

The tensile properties of the RC and PLA membranes were tested according to ASTM D638-10 using an Instron 5966 (Instron, Boston, MA, USA) tensile testing machine mounting a load cell of 250 N. Test specimens of 75 × 10 mm dimensions, after conditioning at ambient conditions for a day, were used for a gauze length of 30 mm for the test. The samples were stretched at a crosshead speed of 10 mm min^−1^. Five replications were tested for each sample. The typical stress–strain curves were plotted from the measured load and extension vales. A digital caliper (Electron Microscopy Sciences, Hatfield, PA, USA) was used to measure the thickness of the specimens.

#### 2.2.8. Contact Angle, Surface Energy, and Wetting Envelope

The static water contact angle measurements were performed at room temperature following the guidelines of ASTM-D7334-08, sessile drop method, by a video-based drop shape analyzer (OCA 25, Data Physics GmbH, Regensburg, Germany). A droplet of 2 μL of deionized water was dropped from a micro-syringe to the sample surface. The contact angles were measured by the built-in SCA20 software (V.4.5.20). For each sample, the contact angle was measured on three different membranes, and an average of three readings was reported.

The dynamic contact angle measurements, advancing and receding contact angles, were performed at room temperatures following the Wilhelmy plate technique using a tensiometer (DCAT 11, Data Physics GmbH, Regensburg, Germany) interfaced with a computer using DCATS software (V.4.1.14). The measurements were taken at room temperature at a 0.2 mm s^−1^ motor speed. The tensiometer measured the total force exerted on the membranes during immersion (immersion depth 3 mm) in the probe liquids. The reported measurements are the average of three readings. 

The OWRK method was used to calculate the total surface energy (SFE) and its polar and dispersive components [25] using the contact angles measured in at least two probe liquids following Young’s [4] equation in Equation (1).

(1) $$\gamma_{M}=\gamma_{ML}+\gamma_{L}Cos\theta ,$$

where $\gamma_{M}$ is the SFE of the solid membranes; $\gamma_{L}$ is the surface tension of the liquid; $\gamma_{ML}$ is the interfacial SFE of the membrane–liquid interface; and θ is the dynamic contact angle. The interfacial SFE between a solid surface and a liquid is calculated following the OWRK equation using a liquid of known surface tension components.

(2) $$\gamma_{ML}=\gamma_{M}+\;\gamma_{L}-2\sqrt{\gamma_{M}^{dispersive}\gamma_{L}^{dispersive}}\;-2\sqrt{\gamma_{M}^{polar}\gamma_{L}^{polar}},$$

where $\gamma_{M}^{dispersive}$ and $\gamma_{M}^{polar}$ are the dispersive and polar components of the surface energy of the solid surface; $\gamma_{L}^{dispersive}$ and $\gamma_{L}^{polar}$ are the dispersive and polar components of surface tension of the test liquid. This equation is modified to a working formula (Equation (3)) by substituting some of the expression from Young’s Equation (1) in the OWRK Equation (2).

(3) $$\gamma_{L}\left(1+Cos\theta \right)=2\sqrt{\gamma_{M}^{dispersive}\gamma_{L}^{dispersive}}+2\sqrt{\gamma_{M}^{polar}\gamma_{L}^{polar}},$$

(4) $$\frac{\;\gamma_{L}\left(1+Cos\theta \right)}{2\;\sqrt{\gamma_{L}^{dispersive}}}=\sqrt{\gamma_{M}^{polar}}*\;\sqrt{\;\frac{\gamma_{L}^{polar}}{\gamma_{L}^{dispersive}}}\;\;\;+\sqrt{\gamma_{M}^{dispersive}}.$$

The expression (4), a modified form of (3), is the expression of a linear equation (y = mx + c) where the values of y and x are known. The values of the polar and dispersive components of SFE are contained in the value of m (slope) and c (axis intercept), respectively. A regression line with two test liquids can be used to obtain the components of the SFE from which the total SFE is calculated following Equation (5). Wetting is favored by a low liquid surface tension, low interfacial SFE, and high solid SFE [25].

(5) $$\gamma_{M}^{total}=\gamma_{M}^{dispersive}+\gamma_{M}^{polar}.$$

The surface energy of the samples was calculated in DCATS software using the average values of advancing and receding contact angles (*θ*) from three readings. Water, formamide, and glycerol have been used as non-solvents for surface energy calculation following the Owens and Wendt method [25]. The surface tension values of the liquids taken from the software are listed in Table 3. The wetting envelopes, a 2D map of wetting, of the membranes were constructed by plotting the polar and dispersive components of the SFE against each other.

## 3. Results and Discussion

### 3.1. Fabrication of Electrospun Nanofibrous Membranes 

**Figure 2:**
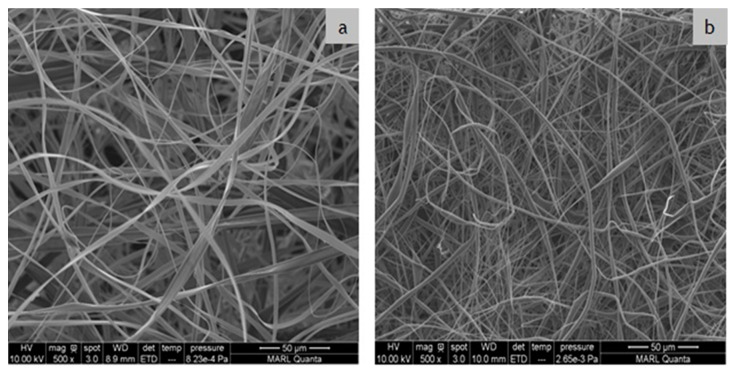
SEM images of as-spun (**a**) CA and (**b**) PLA membranes fabricated on copper foil.

The fabrication of PLA and CA ENF membranes using the electrospinning technique is critical, and requires precise control over the processing parameters. A combination of polymer and solvent properties, including the polymer molecular weight, solution concentration, viscosity, surface tension, conductivity, vapor pressure, and solubility, have great influence on the development and morphology of ENFs [26,27]. During our preliminary tests, we have manipulated the electrospinning parameters to find a suitable solvent system and optimum processing conditions. Spinning fluid with a CA mass concentration of less than 15% in acetone could not form bead-free continuous fibers due to the lower viscosity of the solution and insufficient polymer chain entanglement. At concentrations of less than 10% (*w*/*v*) electrospraying occurred instead of electrospinning. A CA concentration below 15% (*w*/*v*) exhibited fibers with beads. Therefore, the CA fibers were electrospun at a 15% (*w*/*v*) concentration in pure acetone. Similarly, after numerous trials with different binary and ternary solvent systems, an 8% (*w*/*v*) solution of 1:3 chloroform:acetone was used for electrospinning bead-free PLA fibers. The SEM images of the as-spun CA and PLA ENF membranes are presented in Figure 2. Both the CA and PLA ENF membranes are layers of randomly oriented continuous bead-free fibers with an open porosity and different morphologies.

### 3.2. Confirmation of the Development of Regenerated Cellulose (RC)

**Figure 3:**
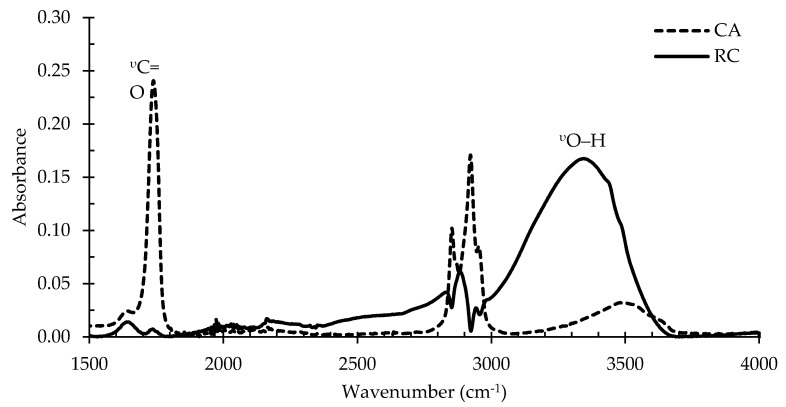
FT-IR spectra of cellulose acetate (CA) and regenerated cellulose (RC) membranes.

FTIR spectroscopy was used to study the formation of regenerated cellulose via the change in the chemical structure of the cellulose acetate ENFs. Changes in the FTIR spectra due to the deacetylation process of CA membranes are displayed in Figure 3. The characteristic absorbance peaks attributed to the vibrations of the acetate group at 1745 (^υ^C=O), 1370 (^υ^C–H_3_), and 1225 cm^−^^1^ (^υ^C–O–C) decreased in intensity or disappeared, indicating the successful conversion of CA into RC [28]. Moreover, the intensity of the broad absorbance peak at 3500 cm^−^^1^ (^υ^O–H) is remarkably increased after deacetylation, indicating the development of a large number of O-H bonds in the RC due to deacetylation [28]. The shift of the O-H band in RC toward a lower wavenumber at 3350 cm^−^^1^ is also an indicator of the formation of a large number of hydrogen bonds in RC [29]. No new peaks were developed after deacetylation. The result is in complete agreement with the published literature on the successful regeneration of cellulose [29].

### 3.3. Size and Morphology of the ENFs

**Figure 4:**
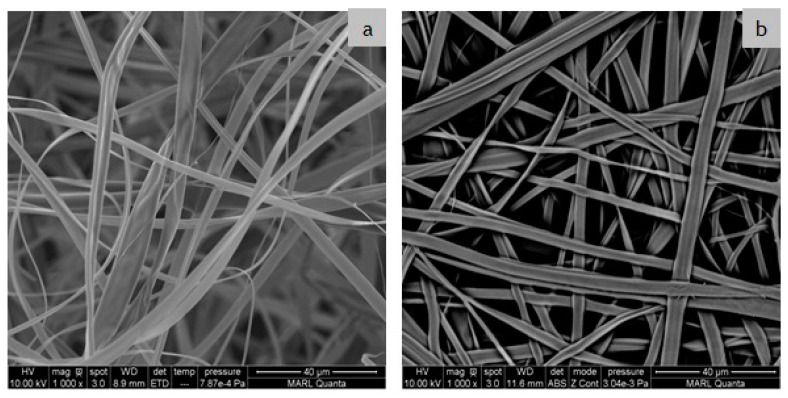
SEM images of as-spun (**a**) CA and (**b**) RC ENF membranes fabricated on copper foil.

The effects of the deacetylation of the CA fibers on the fiber morphology are presented in Figure 4. It is evident from the SEM images that the fibers are bead-free continuous fibers. The as-spun CA membranes were fluffy and showed less adhesion among the fibers due to the presence of the charged acetate group on the fiber surface. Hydrolysis during the deacetylation process greatly affected the morphology of the fibers and resulted in a compact packing due to the increased cohesion among the fibers. This can be attributed to the introduction of additional hydroxyl groups to the fiber due to the deacetylation process, as was seen in Figure 3. In a previous study, Zheng et al. obtained beaded fibers with non-uniform diameters where the individual fibers became closer due to the shrinkage after alkaline treatment and formed a fused morphology [29]. Other researchers have explained this phenomenon as an increase in the structural density and higher alignment of the nanofibers [24,28]. 

**Figure 5:**
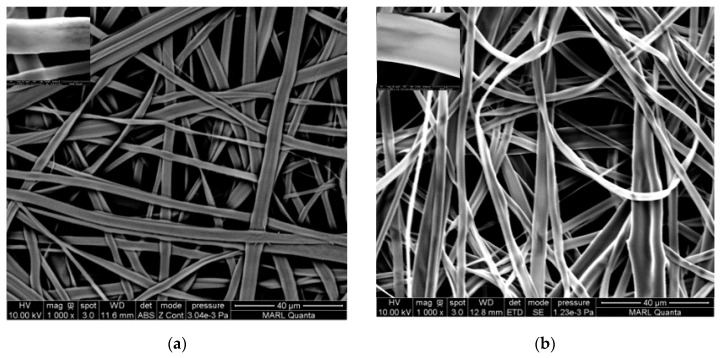

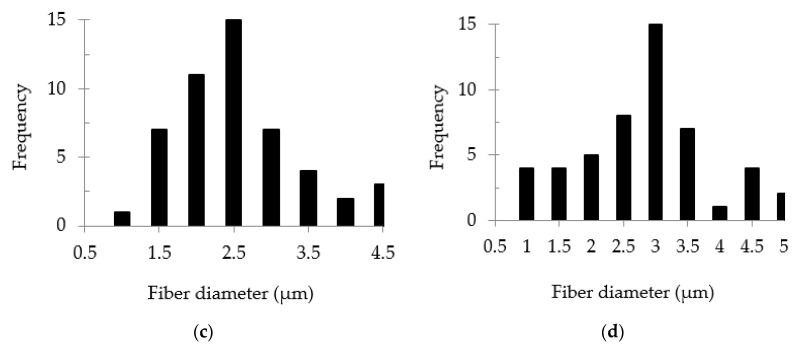
SEM images (1000×) of the RC ENF membranes collected on different collectors: (**a**) copper foil and (**b**) 30-mesh copper gauze. Inset images are fibers at a higher (10,000×) magnification. The histograms below the images, (**c**,**d**), represent the corresponding fiber distributions.

**Figure 6:**
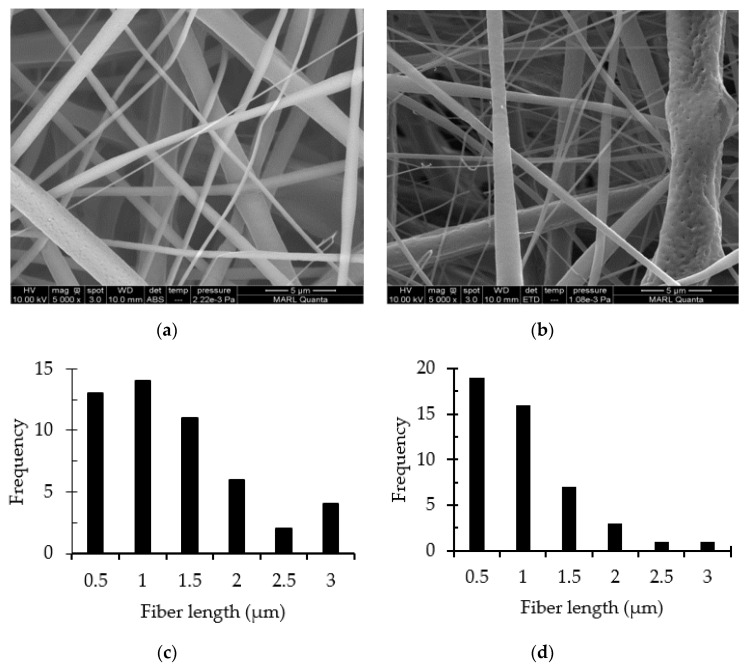
SEM images (5000×) of the PLA membranes collected on different collectors: (**a**) copper foil and (**b**) 30-mesh copper gauze. The histograms below the images, (**c**,**d**), represent the corresponding fiber distributions.

The effect of collectors is presented in Figure 5 and Figure 6, with the respective fiber diameter distribution below the images. The SEM images of the RC fibers in Figure 5 depicts that the morphology was not greatly affected by the collector type. Flat and ribbon-like structures with microscopic pores among the fibers were observed. The average diameter of the RC fibers was measured as 2.5 ± 0.8 and 2.3 ± 0.8 microns when collected on copper foil and 30-mesh copper gauze, respectively. On the other hand, the PLA fibers (Figure 6) exhibited a significant response to the collector geometry; bead-free fibers were obtained when collected on copper foil, but fibers with rough surfaces and bead-like irregularly thick and thin places were observed when collected on the mesh collector. The average PLA fiber diameters were measured as 1.2 ± 0.8 and 0.8 ± 0.7 microns when collected on copper foil and 30-mesh copper gauze, respectively. Consistent with the literature, cylindrical and porous fiber structures were formed in the PLA membranes [12,23]. Wu et al. ascribed the porous structure to the rapid volatilization of the low boiling point solvent system [30].

The wide distribution of the fiber diameters is a common feature of ENFs, especially when highly volatile solvents are used [8]. Most of the histograms of the fiber diameter distribution are non-symmetric and right skewed, meaning the fiber distributions are rich in thinner fibers. Fine fibers were mostly favored by the 30-mesh copper collector. This might be due to the higher electrostatic field intensity at the densely woven copper grids, which induce additional stretching on the polymer jet [31]. Due to having a larger surface area per unit volume, increased surface roughness, and interconnected porosity, the finer fibers are expected to exhibit superior wetting properties [14].

### 3.4. Tensile Properties

The tensile properties of the monolayer ENF membranes were tested to evaluate their feasibility for specific applications such as CPC for pesticide applicators. Table 4 lists the tensile properties of the ENF membranes with thicknesses ~0.10 mm collected on various copper collectors. The mean value and standard deviation were calculated from the measurement of five specimens. 

**Figure 7:**
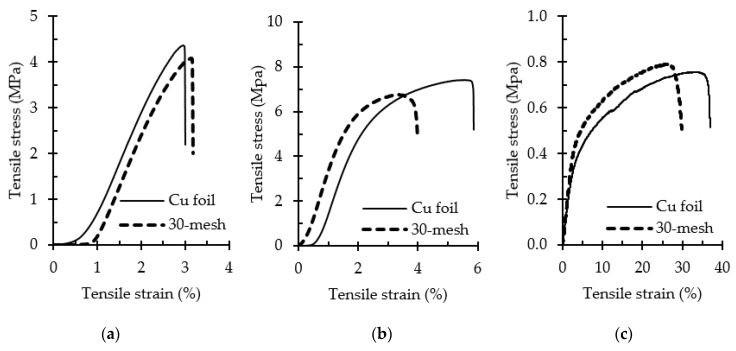
Typical stress–strain curves of (**a**) CA, (**b**) RC, and (**c**) PLA monolayer nonwoven ENF membranes collected on different collectors.

The typical stress–strain curves of the monolayer membranes are plotted in Figure 7. The tensile strength of the CA ENFs (Figure 7a) increased remarkably upon the hydrolysis of CA into RC (Figure 7b) due to the generation of a large number of hydroxyl groups in the structure, as was seen in Figure 3. Moreover, an increase in cohesiveness among the fibers at the crossover points, as was seen in Figure 4, might have also played a role in the increased strength of the RC membranes. Although the collector type did not affect the fiber strength significantly, a correlation between the fiber diameter and tensile strength was observed. Developed on the copper foil collector, thicker fibers exhibited a superior tensile strength and elongation in general due to their ability to withstand a prolonged stretching force [32].

The RC and PLA ENFs exhibited very different tensile strengths, elongations at break, and Young’s moduli. The RC membranes exhibited a tensile strength and elongation compatible with textile structures such as cotton plain cloth [33]. On the other hand, the PLA membranes exhibited a poor tensile strength and Young’s modulus but an exceptionally high elongation. Therefore, we postulated that a combination of RC and PLA membranes in a multilayer structure would complement each other’s tensile properties. Consequently, we investigated the tensile properties of the multilayer structures. The double-layer (RC-PLA) structures exhibited a strength around the average value of strengths of monolayer RC and PLA ENF membranes (Supplementary Materials Figure S1). The multilayer structures exhibited interesting tensile properties; a plateau region was observed, for which the detailed investigation will be reported in our next article. PLA-based triple-layer sandwich structures (PLA-RC-PLA) exhibited a poor tensile strength (Supplementary Materials Figure S2) as compared to the RC-based structures (RC-PLA-RC). The superior tensile strength of the RC-based structures (Supplementary Materials Figure S3) manifests their good fit for textile applications. 

### 3.5. Water Contact Angle 

#### 3.5.1. Static Contact Angle

**Figure 8:**
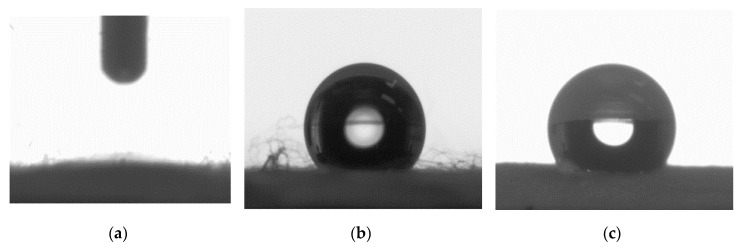
Static water contact angle of the (**a**) RC, (**b**) PLA cu foil, and (**c**) PLA ENF on 30-mesh copper gauge.

The wettability of the RC and PLA membranes was evaluated by contact angle measurements using water as a test liquid. The static water contact angle (SCA) of RC membranes, irrespective of the collector type, was too low to be accurately fitted and determined by the software due to momentary spreading, and therefore was designated to be less than 10° (Figure 8a). On the other hand, the average SCA of PLA membranes collected on copper foil and 30-mesh copper gauze were measured as 135° (±1.3°) and 130° (±2.5°), respectively (Figure 8b,c). However, evaluating the wettability of the multilayer structures using SCA became critical, as it emphasizes the surface properties of the top surface only. Moreover, the surface heterogeneity caused by both the chemical heterogeneity and roughness induces different contact angles on different parts of the same solid surface, which can be captured by the dynamic contact angle (DCA) analyzer. Therefore, for an in-depth understanding of the wetting behavior, DCA analyzer was used.

#### 3.5.2. Dynamic Contact Angle

The average dynamic water contact angles of RC and PLA ENF membranes collected on different collectors are presented in Table 5. The monolayer (control) structures of both the polymers exhibited polymer-specific DCA with little variation due to the collector type. This is because the contact angle of solid surfaces is influenced by physical and chemical factors, including the chemical composition, surface texture (roughness), and surface chemistry (heterogeneity) [34]. A little larger contact angles (~5%) were observed for fibers collected on 30-mesh copper collectors. The surface roughness of the fine fibers collected on mesh collectors, as was seen from the morphology study, can be attributed to this tendency toward hydrophobicity.

Similar to the monolayer membranes, the collector geometry influenced the wettability in multilayer ENF structures; the mesh collectors exhibited larger contact angles than the foil collectors. Among the layered structures, the dual-layer structures exhibited interesting results. Since this structure was created by combining a single RC layer on one side and a single PLA layer on the other side, the resultant DCA was the average of individual contact angles of the membranes (Table 5). The RCs became more hydrophobic in the presence of PLA, while the PLAs became more hydrophilic in the presence of RC in the assembly. Similar results were observed by Prince and Anbharasi when working on multilayer hydrophilic–hydrophobic membrane structures [35].

The triple-layer structures became more hydrophobic in general than control monolayer structures. PLA-based triple-layer structures remained hydrophobic, irrespective of the collector type. RC-based triple-layer structures showed an increased hydrophobicity due to the presence of the sandwiched PLA layer. The dispersive nature of the PLA might have affected the total hydrogen bonding of the structure. Yet, the RC-based triple-layer structures showed a contact angle of 48.2°, which is hydrophilic, and very comparable to the contact angle (47.0°) of the single-layer cellulosic structures [36]. 

### 3.6. Surface Energy and Wetting Envelope

The surface free energy (SFE) is the key parameter beside surface roughness to manipulate the wettability of a solid surface [14]. Therefore, the design of multilayer assemblies must be supported by the corresponding surface energies to predict the wetting behavior. The SFE is a characteristic of a solid surface and is usually determined using two or more non-solvents. Table 6 lists the calculated total SFE of the ENF membrane surfaces collected on various copper collectors.

The collector geometry and layered structure both affected the SFE of the ENF membrane structures. The surface energy decreased significantly in layered structures. This behavior can be related to the surface roughness and amount of hydrogen bonds. According to the theory of Wenzel (1936), an increase in the surface roughness increases the total surface area, and hence influences the calculated SFE and wetting behavior as well.

**Figure 9:**
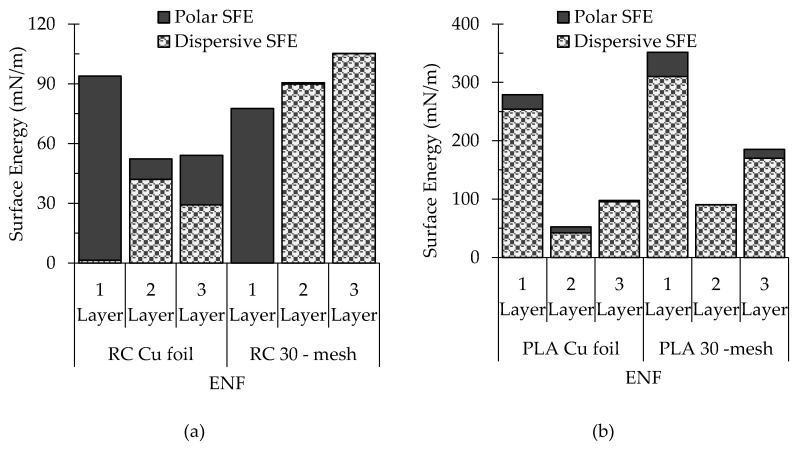
The distribution of polar and dispersive components of surface energy in (**a**) RC-based (**b**) PLA-based structures.

The total SFE of any surface is a combination of its polar and dispersive components. The polar component measures the van der Waals components of surface bonds. The dispersive component measures the London dispersive components. To better understand the mechanism of wetting behavior, the distribution of the polar and dispersive components of the total SFE is presented in Figure 9. The SFE of monolayer RC is primarily polar (hydrophilic), whereas it is mainly dispersive (hydrophobic) in the case of PLA.

A 2D map of wetting, known as a wetting envelope, is constructed by plotting the polar and dispersive components of SFE against each other to visualize the wetting prediction by any liquid to which the membranes might be exposed to [37,38]. Liquids that appear inside the wetting envelope would completely wet the surface; liquids appearing outside the envelope would partially wet the surface. 

**Figure 10:**
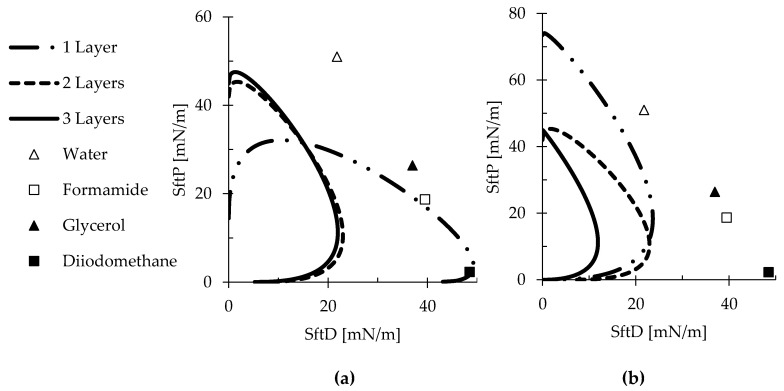
Wetting envelopes of the layered structures of (**a**) RC and (**b**) PLA membranes.

The wetting envelope of both RC and PLA multilayered structures are presented in Figure 10. The polar shift of RC membranes and the contracted PLA wetting envelopes are indicative of unfavorable wetting in multilayer structures. Therefore, wettability can be effectively tuned by creating multilayer sandwich structures consisting of RC and PLA. 

## 4. Conclusions

Two biodegradable polymers, including CA and PLA, were electrospun onto two different copper collectors, namely copper foil and 30-mesh copper gauge. CA ENFs were converted into RC via alkaline treatment. Novel multilayer sandwich structures were created with the RC and PLA ENF membranes only, without any toxic surface treatment. The effect of the collector geometry and sandwich structures on the wettability was studied. Different collectors yielded various sizes and morphologies of the fabricated ENFs with different levels of surface roughness. Finer fibers with an increased roughness were facilitated by the mesh collectors, whereas the foil collectors favored bead-free thicker fibers. Although the fiber strength was not significantly affected by the collector type, a correlation between the fiber diameter and fiber strength was observed. Thicker fibers with a high strength and elongation were obtained when collected on the foil collector. The PLA ENF membranes exhibited a weak tensile strength and low Young’s modulus but exceptionally high elongation. In contrast, the RC ENF membranes exhibited a tensile strength and elongation compatible with textile structures such as cotton plain cloth. Therefore, a combination of RC and PLA ENF membranes in a multilayer sandwich structure would complement each other’s tensile properties. The finer fibers with increased surface roughness, collected on mesh collectors, exhibited increased hydrophobicity due to the higher contact angles and lower surface energies. In multilayer structures, RC-based structures became more hydrophobic in the presence of PLA, while PLA-based structures became more hydrophilic due to the sandwiched RC layer inside the assembly. The RC-based triple-layer structures exhibited a contact angle of 48.2°, which is comparable to the contact angle (47.0°) of the single-layer cellulosic structures. The polar shift of the RC ENF membranes in wetting envelopes are indicative of unfavorable wetting in multilayer structures. This study provides an important insight into the manipulation of the wetting behavior of polymeric ENFs in multilayer structures for applications such as CPC for pesticide applicators.

## Figures and Tables

**Table 1:** Electrospinning design for preparing the CA and PLA ENF membranes.

Polymer	Collector Type
CA	Copper foil
	30-mesh copper gauze
PLA	Copper foil
	30-mesh copper gauze

**Table 2:** Design of multilayer sandwich structures.

Layers	Top Layer	Middle Layer	Bottom Layer	Collector
1	RC RC	----- -----	----- -----	Copper foil 30-mesh copper gauze
	PLA PLA	----- -----	----- -----	Copper foil 30-mesh copper gauze
2	RC	-----	PLA	Copper foil
	RC	-----	PLA	30-mesh copper gauze
3	RC	PLA	RC	Copper foil
	RC	PLA	RC	30-mesh copper gauze
	PLA	RC	PLA	Copper foil
	PLA	RC	PLA	30-mesh copper gauze

Notation: ----- indicates there is no layer.

**Table 3:** Surface tension values of common test liquids.

Liquid	$\gamma_{L}^{dispersive}(\mathrm{mN}/m)$	$\gamma_{L}^{polar}(\mathrm{mN}/m)$	$\gamma_{L}^{total}(\mathrm{mN}/m)$
Water	21.8	51.0	72.8
Formamide	39.5	18.7	58.2
Glycerol	37.0	26.4	63.4

**Table 4:** Tensile properties of the RC and PLA membranes.

ENF Membranes	Collector	Tensile Strength (MPa)	Elongation (%)	Young’s Modulus (MPa)
CA	Copper foil	4.33 ± 0.6	3.2 ± 0.7	247 ± 31
	30-mesh copper gauze	3.83 ± 0.4	2.9 ± 0.6	239 ± 45
RC	Copper foil 30-mesh copper gauze	6.85 ± 0.6 6.75 ± 1.1	6.0 ± 1.7 5.4 ± 1.8	392 ± 71 395 ± 47
PLA	30-mesh copper gauze	0.75 ± 0.1	27.6 ± 6.6	20.2 ± 7.6
	30-mesh copper gauze	0.79 ± 0.2	28.2 ± 6.8	32.6 ± 7.7

**Table 5:** Measured DCA of single and multilayer assemblies.

Dynamic Water Contact Angles (°)				
# of Layers	RC		PLA	
	Copper Foil	30-Mesh	Copper Foil	30-Mesh
1	38.5 ± 1.9	40.2 ± 1.8	91.6 ± 2.9	96.6 ± 1.4
2	63.6 ± 3.4	70.0 ± 2.9	63.6 ± 3.4	70.0 ± 2.9
3	48.2 ± 3.9	72.1 ± 1.5	96.2 ± 2.9	100.1 ± 4.1

**Table 6:** Calculated surface free energy (SFE) of the multilayer structures.

Surface Free Energy (mN/m)				
# of Layers	RC		PLA	
	Copper Foil	30-Mesh	Copper Foil	30-Mesh
1	58.67	56.77	66.23	74.68
2	44.89	48.76	44.89	48.76
3	54.93	50.14	32.33	45.05

## Supplementary Materials

The following are available online at https://www.mdpi.com/2073-4360/12/9/2092/s1, Figure S1: Stress-strain curve of the 2-layers (RC-PLA) ENF structures, Figure S2: Stress-strain curve of the PLA based 3-layers (PLA-RC-PLA) ENF structures, Figure S3: Stress-strain curve of the RC based 3-layers (RC-PLA-RC) ENF structures.
