# Supplementary material for: Tunable Wettability of Biodegradable Multilayer Sandwich-Structured Electrospun Nanofibrous Membranes
Source: Polymers (Basel). 2020 Sep 15;12(9):2092. doi: 10.3390/polym12092092 (PMC7569968; doi:10.3390/polym12092092)
Supplement: Supplementary file 1 [file polymers-12-02092-s001.pdf]

Tunable wettability of biodegradable multilayer sandwich-structured electrospun nanofibrous membranes

AKM Mashud Alam, Elena Ewaldz, Chunhui Xiang,*, Wangda Qu and Xianglan Bai

Supporting Information

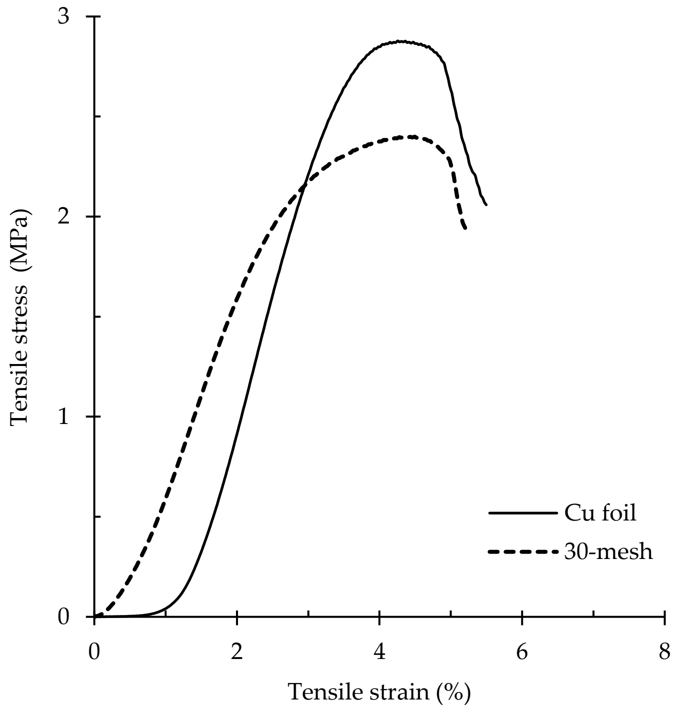

Figure S1. Stress-strain curve of the 2-layers (RC-PLA) ENF structures

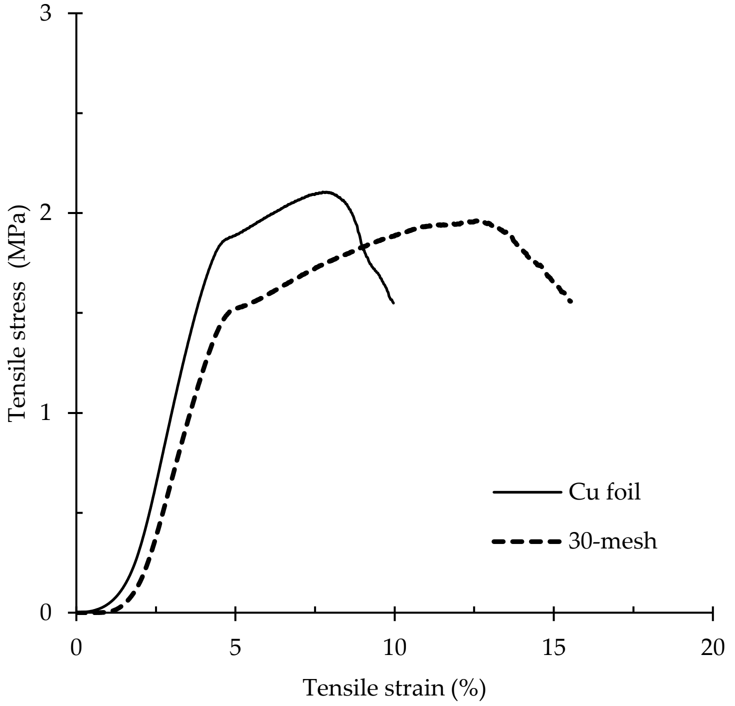

Figure S2. Stress-strain curve of the PLA based 3-layers (PLA-RC-PLA) ENF structures

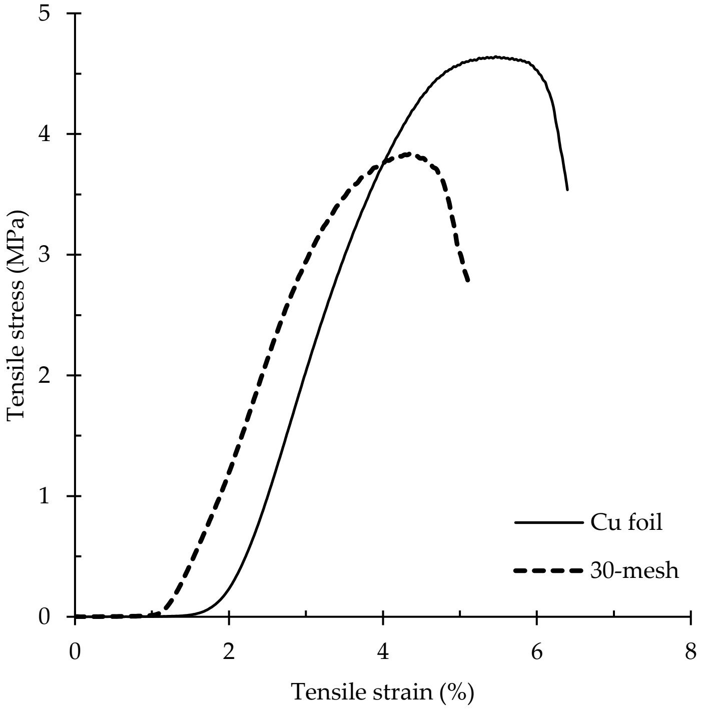

Figure S3. Stress-strain curve of the RC based 3-layers (RC-PLA-RC) ENF structures
